# Keeping your lab together in the COVID-19 era

**DOI:** 10.1186/s13059-021-02457-1

**Published:** 2021-08-19

**Authors:** Timothy R. Sands

**Affiliations:** Springer Nature, London, UK

The COVID-19 pandemic has had a dramatic effect on lives around the world, with scientists and the conduct of their research being no exception. With restrictions in many parts of the world being gradually relieved, we canvassed members of our Editorial Board for their experiences, advice and lessons learned on keeping your lab going, and getting your research up and running again.

We expected to see a difference between groups conducting purely computational work and those with predominantly wet labs, with the former intuitively seeming more amenable to remote working. While one fortunate respondent indicated that their computational lab functioning had continued without major issues arising from home working, this was an exception. Computational researchers found themselves just as vulnerable to the effects of isolation and difficulties from lack of face-to-face collaboration as their wet-lab colleagues. Indeed, a common theme emphasized in the majority of all responses was the importance of helping colleagues with their mental wellbeing, maintaining lab morale, and facilitating return to the lab in person.

Ross Fitzgerald said, “Many people suffered psychologically from the enforced isolation allied to concern regarding lack of progress in their science, particularly those working towards post-graduate degree. My computational scientists have been less affected with regard to progress but of course they have had similar mental challenges to the others- they’ve kept pretty positive though… the biggest challenge has been managing my group/keeping spirits up from afar- I put aside all of Monday for lab meeting and one-to-one catch-ups with everyone in my group.”

Sam Aparicio raised similar points: “We found that careful attention to regular interactions, not all based around work matters, was important to keep cohesion and a sense of community and purpose.” … “The general assumption has been that dry lab working is less affected, but this is not entirely the case. Some persons don’t have conducive home environments, others have found that the complete absence of social contact in the environment to lead to a loss of structure. We allowed part time work by informaticians and admin staff from last quarter of 2020, because it was important for social cohesion.”

Phillippe Collas agreed: “Anticipate the psychology of staying home for weeks, whether this is with small children, or alone. Follow up with individuals more closely: more frequent contacts, socially-distanced socializing outside, etc.”

Within the specific boundaries set by national and institutional restrictions on return to work in person, there was advice on getting lab members back to working together. Phillippe Collas has advice for encouraging reluctant colleagues: “Getting people back to the lab or their offices has not been translated into major enthusiasm. Despite the reopening, I have experienced challenges. People seemed to appreciate working from home, avoiding even short commutes (by public transport, even when commuting in rush hour could easily be avoided). It’s as if home had become the default. Paradoxically, I have experienced that some young people working from home the whole Spring and Summer had a hard time coming back to the lab or office, and felt “unwell” or on-and-off “sick”: gentle yet persistent argumentation for why “coming back to the lab would be good for you” seemed to, however, work – to a point where “sickness” became a non-issue after a few days.”

Fowzan Alkuraya saw a parallel with existing trends, pointing to the importance of a return to the lab in person: “I recall once hearing a renowned senior researcher in neurogenetics remark that he is seeing a trend over the recent years with lab members spending more time on their computers than at the bench. It is true that combined availability of big data and powerful computational tools have enabled once unimaginable research activities. However, the fact remains that our field will always rely heavily on experimental data generated in the wet lab. The recent experience with the COVID19-related lockdown brought this into sharp focus when labs around the world realized that there is only so much one can accomplish remotely.”

Working and communicating online has taken a much increased role in many of our lives, and this seems likely to a feature of much future working. Our respondents have advice for making the most of online interactions.

Christoph Bock says, “While we have of course switched all large seminars to video conferencing, we have maintained the option for some small meetings and even some teaching to occur in person. This applies specifically to highly interactive formats, brainstorming sessions, and mentoring meetings. Thanks to the wide availability of masks (initially surgery masks, now always FFP2 masks) and additional safety precautions (such as big rooms, ventilation or open windows, and rapid antigen tests for guests who are not part of our usual testing routine), the associated risks appear minimal, and the perceived benefits for scientific and personal progress of students/postdocs is high.”

The mental toll of video conferencing was noted by Sam Aparicio: “Learning to use Zoom was important. We found that short Zooms, 30-45 minutes, with enforced breaks of at least 15 minutes between Zooms were essential to avoid burnout.”

Physically getting back into the lab posed difficulties for some, as did what to prioritize once there. Phillippe Collas notes as an Institute head, “A major challenge has been to interpret University regulations upon partial re-opening. “Critical experiments” were again allowed, and decisions on how was allowed to come back to the lab were to be made by the heads of institute and department. But based on what? The new regulations required group leaders to literally apply for every person to come back, providing details on rooms, days, time of day, activity, etc. etc. A bureaucratic nightmare. And how were we, heads of institute and departments, to judge the essence of an experiment? In short, we ended up granting over 90% of the requests. After the fact, what could we have done differently? Simplify the “application process” to a minimum for re-entering the building, as it remained largely empty. and not react so strongly and swiftly to a shutdown (e.g. by throwing cell cultures away): it turns out it would have been possible (and allowed) to come back to finish off experiments or safely put way materials, even after shut down.”

Even once back in the lab, new sources of delay in experiments have emerged, with shortages of materials and negative changes to funding hampering many in their return to normal working. Ami Bhatt notes that, “It really has been tough - especially with the most recent MAJOR plastic shortages. We haven't been able to access adequate pipet tips/plates to even complete experiments that are mid-flight... a very, very difficult time.” Phillippe Collas agrees, “Throughout Fall 2020 and Spring 2021, delays in deliveries, and shortage of reagents / lab supplies, are increasing. This obviously hampers research progress.”

As a result, researchers are urging editors and reviewers to show understanding, and authors are recommended to communicate their difficulties with editors about delayed or currently impractical revisions. Kin-Fai Au says, “It is key to establish good communication with editors, when the authors are planning the experiments and data collection to response the reviewers' comments. For example, upon receiving the revision request, the authors may discuss with editors for the priority of experiments and data collection and settle a list of “must do.” Considering the possible shutdown and the restricted laboratory capacity, the authors are strongly encouraged to provide in advance the editors a time estimate of completing the essential experiments and data collection.”

Sam Aparicio says, “My advice on revisions is that there is not much to be gained at present by enforcing time taken for revisions and some degree of consideration as to what is really essential and what is nice to have, should be exercised, the need to make conclusions robust notwithstanding. Labs are not generally functioning at full efficiency, distance working, institute closures, supply chain issues with reagents, loss of research funding all due to pandemic effects, have affected many labs. The loss of research funding will be a growing paradoxical effect of the pandemic. Certain sectors are prospering; others such as cancer research, anything supported by charities that fundraise from the public, are being hammered.”

However, despite the many problems arising from the pandemic, respondents saw silver linings in all the clouds, and even development opportunities, free from some of the pressures of normal times. Fowzan Alkuraya has several recommendations: “That is not to say of course that labs are doomed to non-productivity during lockdowns. To the contrary, these should be viewed as advantageous “downtimes” that can be turned into precious opportunities. One obvious advantage is the ability to reassess and reflect on the lab priorities. On a more practical note, these are precisely the times that lab members and the principal investigator can utilize for the actual writing of manuscripts that may have been put off during the busy day-to-day lab activities. A thorough review and cleanup of the lab’s digital resources are always on the to-do list but get bumped by more urgent lab priorities. A review article that engages lab members is another great “downtime” activity. If nothing else, what could be a better time to finally get to read all those articles and book chapters that you were too busy to read?”

Kin-Fai Au notes, “As bioinformatics and statistical analyses become prevalent in biomedical research, the wet-lab team, including PIs, postdocs and students, are highly encouraged to learn these skills with tremendous online materials when laboratory capacity is restricted.”

And one final note of encouragement from Kelly Frazer, “I think advice is going to be specific to the institution where the PI works. It will be much harder for labs that fully shut down. I am not sure what advice I could give PIs other than we have to get the scientific enterprise back up to speed, so roll up our sleeves and get to it.”

